# Prevalence of Congenital Heart Disease in Xinjiang Multi-Ethnic Region of China

**DOI:** 10.1371/journal.pone.0133961

**Published:** 2015-08-28

**Authors:** Fen Liu, Yi-Ning Yang, Xiang Xie, Xiao-Mei Li, Xiang Ma, Zhen-Yan Fu, Bang-Dang Chen, Ying Huang, Chun-Fang Shan, Yi-Tong Ma, Xiao-Ming Gao

**Affiliations:** 1 Xinjiang Key Laboratory of Cardiovascular Disease, Clinical Medical Research Institute, The First Affiliated Hospital of Xinjiang Medical University, Urumqi, China; 2 Department of Cardiology, First Affiliated Hospital of Xinjiang Medical University, Urumqi, China; 3 Baker IDI Heart and Diabetes Institute, Melbourne, Australia; Bambino Gesù Children's Hospital, ITALY

## Abstract

**Background:**

The prevalence and risk factors of congenital heart disease among Xinjiang, northwestern part of China is currently unknown.

**Methods:**

This multiple-ethnic, community-based, cross-sectional study was conducted to estimate the prevalence and distribution of congenital heart disease (CHD) in Xinjiang, northwestern part of China. Four major ethnics, Uygur, Han, Kazak, and Hui children in this region were investigated during February 2010 and May 2012.

**Results:**

A total of 14,530 children (0–18 yr) were examined. Of these children, 240 (boys, 43.8%, and girls, 56.3%) were identified with CHD, giving an overall prevalence of 16.5‰ (17.7‰ in Uygur, 6.9‰ in Han, 11.4‰ in Kazak, and 38.1‰ in Hui Chinese, respectively). Ventricular septal defect (VSD, 29.2%), atrial septal defect (ASD, 20.8%), patent ductus arteriosus (PDA, 13.7%), acleistocardia (13.7%), Bicuspid aortic valve (7.9%), pulmonary valve stenosis (5.4%), and tetralogy of fallot (TOF, 4.2%) were common cyanotic and cyanotic defects observed. Compared to non-CHD children, children with CHD had a higher percentage of history of abortion, CHD history of family, consanguinity and premature birth (*all P<0*.*05*). In CHD children, 24% of mothers caught a cold, 10% had a febrile illness and 6.7% received antibiotic treatment during the first trimester of pregnancy, that were higher than non-CHD group (*all P<0*.*05)*.

**Conclusion:**

The overall prevalence of CHD in four ethnic children at ages 0–18 yr in Xinjiang was 16.5‰. VSD, ASD and TOF were the most common acyanotic and cyanotic congenital heart defects, respectively. This study also identified some modifiable risk factors that may contribute to the incidence of CHD among the 4 ethnic groups.

## Introduction

Congenital heart disease (CHD) is one of the most common congenital defects and accounts for nearly one-third of all major congenital anomalies [1]. Some population-based epidemiological studies on CHD have indicated a prevalence ranging from 4 to 50 per 1,000 live births [2–4] and the incidence is even higher in cases of premature children, stillbirth or spontaneous abortion [5]. CHD in children continues to be an important cause of death [6], and constitutes a potential risk of sudden cardiac death in adulthood even with mild cardiac lesion [7]. With the development of diagnostics and cardiothoracic surgery and the introduction of intracardiac interventional techniques, survival of affected children has markedly improved, which have continuously influenced the size of the population of patients with CHD [6]. An analysis study based on the China Ministry of Health 2003 to 2010 annual reports demonstrated that the overall mortality rate of CHD increased from 141 per 10,000,000 person-years in 2003 to 229 per 10,000,000 person-years in 2010, a 62.4% relative increase [8]. According to *China Daily* report, more than 300,000 children are born each year with CHD in the mainland of China, only 70,000 of them have access into medical treatment [9]. In Xinjiang, a remote multi-ethnic region is a less developed area compared with Central and East parts of China, estimated incidence of CHD are higher than the average of national levels and less CHD children receiving proper medical treatment. Further, variation or inaccuracy of epidemiological data in the prevalence of CHD also prevents appropriate distribution of limited medical resources.

In 2010, the government of Xinjiang launched a medical assistance project aiming to provide free medical treatment to children with CHD. To gain the information on the prevalence of CHD in Xinjiang, China, a community based epidemiological investigation was prospectively conducted in 3 representative regions covered major ethnicities in Xinjiang from 2010 to 2012.

## Methods

### Ethics Statement

This study was approved by the Ethics Committee of the First Affiliated Hospital of Xinjiang Medical University (Xinjiang, China). It was conducted in accordance with the standards of the Declaration of Helsinki. As all participants were children and adolescents, the written informed consents were obtained from parents of all participants.

### Subjects

We used a random sampling method to select children (0–18 yrs of age) from a community-based population covered four major ethnicities, i.e. Uygur, Han, Kazak and Hui Chinese in Xinjiang. Three representative regions (Hetian, Aletai and Kashi) were chosen and one child was randomly selected from each household based on the government record of registered residence. Children were selected from 31 natural villages and residential areas and invited to participate. For those whose data were incomplete or disagreed to participate were excluded.

### Collection of history, survey and medical examination

Collection of family or clinical history and survey were carried out using a standard questionnaire. Each child received general and cardiovascular examinations by a pediatric cardiologist. CHD was suspected in the presence of a cardiac murmur, cyanosis, clubbing, features of congestive heart failure and failure to thrive. Children showing any of these abnormal conditions would receive 12-lead electrocardiography and echocardiography. Echocardiography was conducted using Acuson Cypress cardiac portable ultrasound machine (SIEMENS, Germany). Two-dimensional echocardiographic traces and standard images from parasternal long axis, short-axis, apical four chamber, subcostal and suprasternal views were obtained and analyzed by cardiologists. CHD was diagnosed and confirmed if any structural abnormality of the heart or intrathoracic great vessels presented regardless whether there was potential functional significance, as defined previously [10]. In addition, children with cardiac arrhythmias or patent ductus arteriosus in premature newborns less than one month of age were not diagnosed as CHD and were excluded.

### Data management and statistical analysis

All data from the questionnaire were double-entered and cross-validated using EpiData version 3.1 (EpiData Association, Odense, Denmark). Statistical analyses were performed in SPSS version 17.0 (SPSS Institute, Chicago, IL, USA). Continuous variables are expressed as mean±SD, categorical variables as percentages and exploratory data analysis was performed using descriptive measures. Chi-square test (χ2) was used to detect associations in categorical data and for identifying differences in prevalence. Potential risk factors for CHD were analyzed using a multivariate unconditional logistic regression. *P<0*.*05* was regarded as a statistic significance.

## Results

### Characteristics of study participants

In total, 14,530 individuals (7,183 boys and 7,347 girls) with the median age of 9.9±2.6 yrs (10 days to 18 yrs) were included and analyzed in the present study(S1 Table). According the distribution of different ethnicities in the 3 regions, the majority of participants were Uygur followed by Han, Kazak and Hui children. The basic characteristics of study participants were shown in Table 1. Body weight in Uygur was lower than other 3 ethnic groups, but the prevalence of hypertension in Uygur was higher then other 3 ethnic groups. There were no difference in age, blood pressure and other anthropometric parameters and diabetes among the 4 ethnicities. We found one of CHD child (Uygur) had renal dysplasia (Table 1).

**Table 1:** General characteristics of the project participants from 4 ethnic groups.

	Uygur n = 10448	Han n = 2014	Kazak n = 1401	Hui n = 657
Age, yr	9.9±2.5	9.6±3.5	10.6±2.8	10.7±2.7
Height, cm	133±14	139±19	144±16	145±16
Body weight, kg	30.1±8.2*	35.4 ±13.4	38.9±12.3	39.1±13.7
BMI, kg/m^2^	16.7 ±2.3	17.7± 3.0	18.2± 2.6	18.1±3.2
Heart rate, bpm	90±15	90±16	88±15	87±15
SBP, mmHg	95±15	94±11	96±9	95±8
DBP, mmHg	58±13	59±9	60±6	60±5
*Comorbidity*				
Diabetes, [n (%)]	27 (0.2)	4 (0.2)	2 (0.2)	0 (0)
Hypertension, [n (%)]	210 (2.0)*	13 (0.6)	1 (0.1)	3 (0.5)
Renal dysplasia, [n (%)]	1 (0)	0 (0)	0 (0)	0 (0)

Continuous variables are presented as mean±SD. BMI, body mass index; SBP, systolic blood pressure; DBP, diastolic blood pressure.

* *P<0*.*01* vs. other 3 ethnic groups.

### Prevalence of CHD

As shown in Table 2, congenital heart defects were identified in 240 children from 14,530 participants, giving an overall prevalence of 16.5 per 1000 children with 14.6‰ in boys and 18.4‰ in girls. The incidence of CHD was 17.7‰ in Uygur, 6.9‰ in Han, 11.4‰ in Kazak and 38.1‰ in Hui children, respectively.

**Table 2:** Prevalence and gender and age distribution of congenital heart disease (CHD) in 4 ethnic groups.

	Uygur		Han		Kazak		Hui		
Age, yr	n	CHD [n (‰)]	n	CHD [n (‰)]	n	CHD [n (‰)]	n	CHD [n (‰)]	*χ2*, *P value*
***Boys***									
0–6	571	28 (49.0)*	214	2 (9.3)	40	1 (25.0)	27	4 (148.1)*	*0*.*001*
7–12	3799	37 (9.7)	625	3 (4.8)	468	4 (8.5)	184	3 (16.3)	*0*.*217*
13–18	733	15 (20.5)	223	0 (0)	194	5 (25.8)*	105	3 (28.6)*	*0*.*044*
*χ2*, *P value*		*0*.*001*		*0*.*154*		*0*.*282*		*0*.*062*	
***Girls***									
0–6	599	45 (75.1)*	175	2 (11.4)	57	0 (0) †	31	0 (0)	*0*.*773*
7–12	4163	43 (10.3)	564	7 (12.4)	426	3 (7.0)	209	10 (47.8) ‡	*0*.*006*
13–18	583	17 (29.2)*	223	0 (0)	216	3 (13.9)	101	5 (49.5)*	*0*.*044*
χ2, *P* value		*0*.*001*		*0*.*202*		*0*.*252*		*0*.*339*	
***Both boys and girls***									
0–6	1170	73 (62.4)*	389	4 (10.3)	96	1 (10.4) †	58	4 (68.9)*	*0*.*016*
7–12	7962	80 (10.0)	1189	10 (8.4)	894	7 (7.8)‡	393	13 (33.1)‡	*0*.*004*
13–18	1316	32 (24.3)*	445	0 (0)	410	8 (19. 1)*	206	8 (38.8)*	*0*.*004*
*χ2*, *P value*		*<0*.*001*		*0*.*029*		*0*.*210*		*0*.*390*	
**Total**	10448	185 (17.7)	2024	14 (6.9)	1401	16 (11.4)	657	25 (38.1)	*<0*.*01*

*P<0.*05* vs. Han in the same age group

†*P<0*.*05* vs. Uygur in the same age group

‡P<0.05 vs. other age groups in the same gender.

### Sex and age distributions of CHD in 4 ethnic children

In this study population, 240 children were diagnosed with CHD and the median age of CHD children was 8.0±4.6 yrs. Of these children, 105 (43.8%) were boys and 135 (56.3%) were girls, given the ratio of boy to girl was 0.78 (S2 Table). There was a trend of a higher detecting rate of CHD in girls in Uygur, Han and Hui children except Kazak children (Table 2). Compared to other ethnic groups, incidence of CHD in Hui children (both boys and girls) was higher. The incidence of CHD varied cross the 3 designated age groups in the 4 ethnicities. Chi-square test analyses showed statistical differences in boys of 0–6 and 13–18 yrs age groups and girls of 0–6, 7–12 and 13–18 yrs age groups or in all 3 age groups of all children with CHD among the 4 ethnicities with a highest incidence in Hui children at 0–6 yrs age group (Table 2). In regards to the incidence of CHD in 3 different age groups in each ethnicity, only Uygur and Han children (both boys and girls) showed a statistical difference (Table 2). Children in 0–6 yrs age group had a relative high incidence of CHD than other 2 age groups in Uygur, Han and Hui ethnic groups except Kazak children.

### Pattern of CHDs

The pattern of CHD observed is depicted in Table 3. Ventricular septal defect (VSD), atrial septal defect (ASD), patent ductus arteriosus (PDA) and acleistocardia were the most common acyanotic congenital heart lesions in all 4 ethnic groups, counting for 29.2%, 20.8%, 13.7% and 13.7% in total CHD cases, respectively, compared to 7.9% bicuspid aortic valve (BAV) and 5.4% pulmonary valve stenosis (PVS). Tetralogy of fallot (TOF, 4.2%) was the most common cyanotic congenital heart lesions. Transposition of the great arteries (TGA, 1.3%), mitral valve prolapsed (MVP, 1.7%), dextrocardia (0.8%) and complex anomaly (1.3%) were the infrequent congenital heart lesions detected. There was no statistical difference in all detected defects among the 4 ethnic groups. In regards to the prevalence of the most common type of CHD in four ethnicities, it was VSD in Uygur (35.1%), PDA in Han (28.6%), and ASD in both Kazak (43.8%) and Hui children (36%). Compared to other 3 ethnic groups, Hui children had a lowest incidence in severe heart lesions without any cyanotic CHD being detected. The majority of CHD detected was acyanotic pattern with 92.4% in Uygur, 78.6% in Han, 93.7% in Kazak and 100% in Hui children and the rest was the cyanotic pattern. The median age of all children with acyanotic CHD was 8.7±4.0 yrs, with cyanotic CHD was 7.4±5.1 yrs. Kazak children with CHD were older than other ethnic groups and Han children with cyanotic CHD was younger than others.

**Table 3:** Pattern of congenital heart disease (CHD) and age in 4 ethnic groups.

Pattern	Uygur n = 185	Han n = 14	Kazak n = 16	Hui n = 25	Total n = 240
Ventricular septal defect, [n (%)]	65 (35.1)	2 (14.3)	3 (18.8)	0 (0)	70 (29.2)
Atrial septal defect, [n (%)]	32 (17.3)	2 (14.3)	7 (43.8)	9 (36.0)	50 (20.8)
Patent ductus arteriosus, [n (%)]	22 (11. 9)	4 (28.6)	1 (6.3)	6 (24.0)	33 (13.7)
Acleistocardia, [n (%)]	27 (14.6)	1 (7.1)	1 (6.3)	4 (16.0)	33 (13.7)
Bicuspid aortic valve, [n (%)]	19 (10.3)	0 (0)	0 (0)	0 (0)	19 (7.9)
Pulmonary valve stenosis, [n (%)]	3 (1.6)	1 (7.1)	3 (18.7)	6 (24.0)	13 (5.4)
Mitral valve prolapse, [n (%)]	3 (1.6)	1 (7.1)	0 (0)	0 (0)	4 (1.7)
Tetralogy of fallot, [n (%)]	8 (4.3)	1 (7.1)	1 (6.3)	0 (0)	10 (4.2)
Transposition of the great arteries, [n (%)]	3 (1.6)	0 (0)	0 (0)	0 (0)	3 (1.3)
Dextrocardia, [n (%)]	2 (1.1)	0 (0)	0 (0)	0 (0)	2 (0.8)
Complex, [n (%)]	1 (0.5)	2 (14.3)	0 (0)	0 (0)	3 (1.3)
Age in acyanotic CHD, yr *	7.2±4.1	9.8±2.4	11.6±3.9	10.7±2.6	8.7±4.0
Age in cyanotic CHD, yr *	7.3±5.1	4.3±5.8	12.5±1.2	0	7.4±5.1

*Data are means ± SD.

To understand which pathology may allow the children to grow up more than others, we analyzed age difference among the identified CHD diagnosis (Table 4). We found that a high incidence of VSD and mitral valve stenosis was in 0–6 yrs and 7–12 yrs group; the highest incidence of ASD, PDA, BAV, TGA and complex was in 7–12 yrs group; the highest incidence of acleistocardia and TOF was in 0–6 yr group and a high incidence of dextrocardia was in 7–12 yrs and 13–18 yrs groups.

**Table 4:** Age distribution of identified congenital heart diseases.

Pattern	0–6 yr n = 82	7–12 yr n = 110	13–18 yr n = 48
Ventricular septal defect [n (%)]	29 (41.5)	29 (41.5)	12 (17.1)
Atrial septal defect [n (%)]	13 (26.0)	29 (58.0)	8 (16.0)
Patent ductus arteriosus [n (%)]	7 (21.2)	17 (51.5)	9 (27.3)
Acleistocardia [n (%)]	19 (57.6)	10 (30.4)	4 (12.1)
Bicuspid aortic valve [n (%)]	5 (26.3)	11 (57.9)	3 (15.8)
Pulmonary valve stenosis [n (%)]	1 (7.7)	5 (38.5)	7 (53.8)
Mitral valve prolapse, [n (%)]	2 (50.0)	2 (50.0)	0 (0.0)
Tetralogy of fallot, [n (%)]	6 (60.0)	1 (10.0)	3 (30.0)
Transposition of the great arteries, [n (%)]	0 (0.0)	2 (66.6)	1 (33.3)
Dextrocardia, [n (%)]	0 (0.0)	1 (50.0)	1 (50.0)
Complex, [n (%)]	0 (0.0)	3 (100)	0 (0.0)

### Potential risk factors may contribute to the incidence of CHD

To further identify potential factors which may contribute to the risk of CHD, we have made a careful history investigation in all participants with or without CHD via a survey. Results are summarized in Table 5. Compared to non-CHD children, children with CHD had a higher percentage of history of abortion which was across 4 ethnic groups. CHD history of family, consanguinity and premature birth were also higher in CHD participants, especially in Hui children (*all P<0*.*05*). Further, among these CHD children, 24% mothers caught a cold, 10% had a febrile illness and 6.7% received antibiotic treatment during the first trimester of pregnancy, that were higher than non-CHD group (*all P<0*.*05*). Differences in parent and mother age when child born were also observed. There was no significant difference in overall altitude of living area, the height above sea level, among the 4 ethnic groups.

**Table 5:** Comparison of potential risk factors between children with congenital heart disease (CHD) or without CHD (non-CHD) in 4 ethnic groups.

Risk factor	Uygur		Han		Kazak		Hui		Total	
	Non-CHD n = 10263	CHD n = 185	Non-CHD n = 2010	CHD n = 14	Non-CHD n = 1385	CHD n = 16	Non-CHD n = 632	CHD n = 25	Non-CHD n = 14290	CHD n = 240
History of abortion, [n (%)]	706 (6.9)	47 (25.4)*	109 (5.4)	4 (28.6)*	78 (5.6)	4 (25.0)*	42 (6.6)	5 (20.0)*	935 (6.5)	60 (25.0)*
CHD history of family, [n (%)]	205 (2.0)	5 (2.7)	33 (1.6)	0 (0)	29 (2.1)	2 (12.5)*	13 (2.1)	4 (16.0)*	280 (2.0)	11 (4.6)*
Consanguinity, [n (%)]	54 (0.5)	7 (3.8)*	4 (0.2)	0 (0)	9 (0.6)	1 (6.2)	4 (0.6)	3 (12.0)*	71 (0.5)	11 (4.6)*
Premature birth, [n (%)]	304 (3.0)	47(25.4)*	37 (1.8)	0 (0)	2 (0.1)	1 (6.3)*	2 (0.3)	1 (4.0)*	345 (2.4)	49 (20.4)*
Paternal age when CHD child born, yr	28.2±4.2	27.7±6.6	28.1±4.0	29.9±5.8*	28.4±3.9	29.1±3.8	28.7±4.1	26.3±4.9*	28.2±4.1	27.8±6.3
Maternal age when CHD child born, yr	25.7±3.8	23.8±4.4*	25.6 ±3.7	27.7±4.3*	25.8±3.7	27.9±5.4*	26.1±3.7	23.4±3.4*	25.7±3.8	25.9±3.8
Altitude of living area, meter	950±672	949±727	956±678	665±456	963±821	1335±2028	935±599	1127±338	951±687	990±834
*During the first trimester of pregnancy*										
Catching a cold, [n (%)]	1613 (15.7)	48 (25.9)*	227 (11.3)	4 (28.6)	255 (18.4)	2 (12.5)	89 (14.1)	4 (16.0)	2184 (15.3)	58 (24.1)*
Febrile illness, [n (%)]	508 (4.9)	20 (10.8)*	55 (2.7)	0 (0)	66 (4.8)	2 (12.5)	18 (2.8)	2 (8.0)	647 (4.5)	24 (10.0)*
Antibiotic prescription, [n (%)]	222 (2.2)	14 (7.6)*	109 (5.4)	4 (28.6)*	31 (2.2)	0 (0)	5 (0.8)	1 (4.0)	282 (2.0)	16 (6.7)*

*P<0.*05* vs. non-CHD group.

Moreover, multivariate unconditional logistic regression analysis for CHD risk factors was also performed. CHD was considered the dependent variable, and other potential risk factors listed in Table 5 except the age and altitude of living area were independent variables. Using the Han children as a reference as it had the lowest prevalence of CHD, the risk of CHD in Uygur, Kazak and Hui children was 1.02-fold (OR = 2.02, 95% CI 1.07–3.83), 0.92-fold (OR = 1.92, 95% CI 0.74–5.0) and 7.92-fold (OR = 8.92, 95% CI 3.93–20.26), respectively (Table 6). Further, the risk of CHD increased significantly with history of abortion (OR = 5.46, 95% CI 3.63–8.21), consanguinity (OR = 5.5, 95% CI 2.69–11.23), premature birth (OR = 9.45, 95% CI 6.70–13.31), catching a cold (OR = 1.68, 95% CI 1.08–2.62), febrile illness (OR = 6.23, 95% CI 2.18–17.82) and antibiotic prescription (OR = 3.71, 95% CI 1.02–13.58, Table 6).

**Table 6:** Multivariate unconditional logistic regression analysis of risk factors for congenital heart disease.

Pattern	β-value	S.E.	Wald	df	P	OR	95% CI
Ethnicity			32.26	3	0.000		
Han						1	
Uygur	0.70	0.33	4.67	1	0.031	2.02	1.07–3.83
Kazak	0.65	0.49	1.79	1	0.181	1.92	0.74–5.02
Hui	2.19	0.42	27.37	1	0.000	8.92	3.93–20.26
History of abortion	1.70	0.21	66.42	1	0.000	5.46	3.63–8.21
Consanguinity	1.70	0.36	21.90	1	0.000	5.50	2.69–11.23
Premature birth	2.25	0.18	164.27	1	0.000	9.45	6.70–13.31
Catching a cold	0.52	0.23	5.34	1	0.021	1.68	1.08–2.62
Febrile illness	1.83	0.54	11.63	1	0.001	6.23	2.18–17.82
Antibiotic prescription	1.31	0.66	3.94	1	0.047	3.71	1.02–13.58
constant	-6.92	0.53	173.79	1	0.000	0.001	

## Discussion

CHD is the most common cause of major congenital anomalies, forming a major global health burden [11]. According to EUROCAT study which covering 1.5 million annual births in 22 European countries, a total prevalence of major congenital anomalies was 23.9‰ births from 2003 to 2007, while the CHD were the most common non-chromosomal subgroup, at 6.5‰ among all different types of birth defects [12]. In the present study, we detected 16.5‰ overall incidence of CHD in 4 different ethnicities including newborn babies and up to 18 yrs old teenagers in Xinjiang, northwest part of China, located in the central of Asia. which is higher than the prevalence reported in European countries, but similar to the 12–15‰ in Asian countries, such as Qatar [13] and Pakistan [14]. A recent meta-analysis study reviewed 114 papers including a total study population of 24,091,867 live births and reported that during 1995 to 2009 the overall birth prevalence of CHD was 9.1‰. A significant geographical differences was found with the highest birth prevalence of CHD in Asia (9.3‰), followed by Europe (8.2‰) and North America (6.9‰) and the lowest in Africa (1.9‰) [6]. It is recognized that variation in the age and size of study population, study design, inclusion criteria and diagnosis sensitivity have led to a wide range in estimated birth and/or prevalence of CHD reported by different studies [3, 6, 11].

Further, a significant ethnic difference was also observed with the highest incidence of CHD in Hui, followed by Uygur and Kazak and the lowest in Han children. The overall prevalence was higher in girls than in boys, which is consistent with others’ reports [15, 16]. A retrospective study in Canada reported a significantly higher incidence of CHD in females in both children under 18 yrs old (52% vs. 49%) and adults (57% vs. 51%) [15]. Whilst, in India, a similar retrospective study observed higher prevalence of most types of CHD in male children from 0 to 15 yrs age [4]. In the present prospective study, there were differences in the 3 designated age groups among these 4 ethnicities but we did not notice an association between the incidence and age in all 4 ethnicities. While the incidence of CHD in 0–6 yrs age group of Uygur, Han and Hui children was higher than other age groups except Kazak children. A similar finding in the young age group was also observed in other populations in Asia [4, 13]. Although the prevalence of CHD is rising in both children and adults, compared to children, adults have a steady decreased prevalence of CHD along with aging [15].

Among the detected congenital heart defects, VSD is the most common malformation account for 29.2% of 240 CHD cases, which is line with the published range of 21–47% in literature [4, 13, 14, 17]. ASD was the second most common defect comprising 20.8%, and this is consistent with previously published studied in China, India and Pakistan with the frequency of 19–23% [4, 14, 18], but is higher than 6%-13% reported from western countries and other part of China [11, 19]. The rest detected acyanotic CHD were PDA, acleistocardia, BAV, PVS and MVP. Overall acyanotic heart defects consisted of the majority of CHD (92.5%) detected in the present study. Only small proportion of CHD was cyanotic heart defects with 4.2% in TOF then followed by TGA, complex anomalies and dextrocardia (<2%). TOF was the most common cyanotic CHD, and its frequency in our study is comparable with other reports [11, 13, 14, 20]. Moreover, we also observed variations in the incidence of different types of CHD among the 4 studied ethnicities with VSD as the most common CHD in Uygur, PDA in Han, and ASD in Kazak and Hui children. Compared to other 3 ethnic groups, Hui children had the lowest incidence in severe and cyanotic heart defects without any case being detected in the studied population while having the highest prevalence in overall CHD. Although it varies in the most common type of CHD and in the proportion of different types of CHD from study to study, the overall distributions of major types of CHD are similar in different populations [3, 11]. Furthermore, we also observed that a high incidence of identified different CHD varied among 3 designated age groups. Most of cyanotic types of CHD, such as VSD, ASD, PDA, acleistocardia, BAV and PVS can be found in all 3 age groups although with a high incidence in a particular age group. In cyanotic types of CHD, a high incidence of TOF was found in both 0–6 and 13–18 yrs groups, while none of TGA, dextrocardia and complex was found in 0–6 yrs group and complex was only found in 7–12 yrs group. This information may help to understand the influence of different types of CHD in natural survival.

As a prevalent birth defect, CHD can occur from genetic and nongenetic factors [21, 22]. To define potential risk factors contributing to the difference in prevalence of CHD in the 4 ethnic groups, we also collected information on family history, marriage situation, parental age when CHD child born, health condition during pregnancy, etc. Overall, the highest proportions of abortion history and catching a cold during the first trimester of pregnancy were found in children with CHD, and followed by febrile illness and antibiotic treatment during the first trimester; the lowest detecting rates were CHD family history and consanguinity. A high premature birth was also found in CHD children compared to non-CHD children. Multivariate unconditional logistic regression analysis revealed a high risk of CHD in Uygur, Kazak and Hui children with the highest OR 8.92 in Hui children (95% CI 3.93–20.26) compared to Han children, suggesting that genetic factors influence the risk of CHD. Further, history of abortion, consanguinity, premature birth, catching a cold, febrile illness and antibiotic usage are also independent risk factors associated with high incidence of CHD with the highest OR 9.45 (95% CI 6.70–13.31) in premature birth.

A potential association between maternal infections and congenital heart defects has been recognized for a few decades [23, 24]. Catching a cold or febrile illness usually links to virus infection e.g. rubella, and influenza [21]. Febrile illness during the first trimester can lead a 2-fold increase in risk of birth heart defects [25]. Further, maternal administration of certain categories of antibiotics and medications are also found associated with higher risk of CHD in offspring [21]. Numerous studies have observed a close link between metabolic diseases, smoking, exposure to toxic chemicals and stress in mothers during early gestation and the risk of offspring CHD (see comprehensive reviews [21, 26]). This information indicates the maternal health condition, especially in the first trimester of pregnancy, is a critical determinant in the risk of birth heart defects. Moreover, increased parental age may also be a risk factor for CHD, but controversy reports make this association uncertain [21, 26]. As observed in the current study, maternal age when CHD child born in Uygur and Hui was significantly lower than Han and Kazak children, while Uygur and Hui ethnic groups had a higher incidence of CHD. Another important factor is consanguinity, as a genetic contributor to CHD, particularly at first-cousin level and closer [27]. In the current study, Hui children with CHD had the highest percentage from consanguineous marriage compared to other ethnic groups. In Xinjiang, northwest part of China, ethnicities such as Hui, Uygur and Kazak have a unique way of life, especially they are less likely to intermarry with other ethnicities, and therefore the chance of consanguinity is higher in these ethnicities than in Han Chinese. Similar situations are also reported in other Asian populations e.g. Iran and India [28, 29]. In regards to the altitude of living areas, high altitude accompanying with lower atmospheric oxygen tension has led to a higher incidence of PDA and ASD [30]. Failure of lower oxygen tension to constrict the ductus and persistent high pulmonary vascular resistance and subsequent high right heart pressure preventing closure of the foramen ovale are though to be the mechanisms [30]. However, as the altitude of resident areas in the current study is not significantly high, we did not observe an association between the attitude and incidence of identified CHD.

### Study limitations

Due to limitations in resources and study duration, this study was community-based, not population-based; therefore, the size of study population was small, which have led to several weaknesses. First, results from the small sample size and three representative regions may not reflect actual prevalence of CHD in the whole region of Xinjiang, northwest part of China, located in the central of Asia and occupying one sixth of national territorial area. Second, due to the small study population, it makes some key comparisons between studied ethnic groups less significant. Third, patterns of CHD detected in the current study were also limited, which may not elucidate overall picture of incidence and distribution of various types of CHD. However, the questionnaire, examination and diagnosis were carried out by a properly trained research team in accordance with the current medical standard, especially, the 4 studied ethnicities are the major ethnic groups in this region, and the results from this study still provide solid representation in the prevalence of CHD. A large scare and population-based epidemiological investigation is warranted in future.

## Conclusions

This community-based study investigated the prevalence of CHD in 4 major ethnic children (Uygur, Han Kazak and Hui) aged from 0 to 18 yrs in 3 representative regions of Xinjiang, the northwest part of China. The overall prevalence of CHD is 16.5‰. VSD, ASD and TOF are the most common acyanotic and cyanotic congenital heart defects, respectively. The difference in the incidence of CHD among studied ethnic groups indicates a genetic influence in risk of CHD. Moreover, this study also identified some potential modifiable risk factors which may contribute to the incidence of CHD among the 4 ethnicities. These data are useful for delivering and distributing limited medical resources and policy making targeting on this congenital defect.

## Supporting Information

S1 Table General characteristics of the project participants from boys and girls. (DOCX)

S2 Table General characteristics of the congenital heart disease from boys and girls. (DOCX)
