# Supplementary material for: Prevalence of Congenital Heart Disease in Xinjiang Multi-Ethnic Region of China
Source: PLoS One. 2015 Aug 28;10(8):e0133961. doi: 10.1371/journal.pone.0133961 (PMC4552834; doi:10.1371/journal.pone.0133961)
Supplement: S1 Table — (DOCX) [file pone.0133961.s001.docx]

**S1 Table**

**General characteristics of the project participants from boys and girls.**

|  | boys n=7183 | girls n=7347 |
| --- | --- | --- |
| Age, yr | 10.0±2.7 | 9.9±2.6 |
| Height, cm | 135.9±15.8 | 135.4±15.5* |
| Body weight, kg | 32.6±10.5 | 31.6±10.2* |
| BMI, kg/m^2^ | 17.2±2.6 | 16.8±2.5* |
| Heart rate, bpm | 95.8±13.6 | 94.4±13.6 |
| SBP, mmHg | 58.9±11.8 | 58.5±11.8* |
| DBP, mmHg | 89.7±15.6 | 89.3±14.3* |

Continuous variables are presented as mean±SD. BMI, body mass index; SBP, systolic blood pressure; DBP, diastolic blood pressure. * *P<0.05* vs. boys.
