# Supplementary material for: Prevalence of Congenital Heart Disease in Xinjiang Multi-Ethnic Region of China
Source: PLoS One. 2015 Aug 28;10(8):e0133961. doi: 10.1371/journal.pone.0133961 (PMC4552834; doi:10.1371/journal.pone.0133961)
Supplement: S2 Table — (DOCX) [file pone.0133961.s002.docx]

**S2 Table**

**General characteristics of the congenital heart disease from boys and girls.**

|  | boys n=105 | girls n=135 |
| --- | --- | --- |
| Age, yr | 8.2±4.6 | 8.3±4.4 |
| Height, cm | 124.8±32.0 | 130.1±28.6 |
| Body weight, kg | 28.8±15.6 | 30.0±15.5 |
| BMI, kg/m^2^ | 16.8±3.0 | 17.1±2.9 |
| Heart rate, bpm | 94.3±15.7 | 91.1±14.5 |
| SBP, mmHg | 57.4±14.1 | 57.7±12.4 |
| DBP, mmHg | 94.0±18.6 | 95.0±18.9 |

Continuous variables are presented as mean±SD. BMI, body mass index; SBP, systolic blood pressure; DBP, diastolic blood pressure.
